# Probiotic supplementation for neonates with congenital gastrointestinal surgical conditions: guidelines for future research

**DOI:** 10.1038/s41390-022-02087-8

**Published:** 2022-05-03

**Authors:** Shripada Rao, Meera Esvaran, Liwei Chen, Chooi Kok, Anthony D. Keil, Ian Gollow, Karen Simmer, Bernd Wemheuer, Patricia Conway, Sanjay Patole

**Affiliations:** 1 Neonatal Intensive Care Unit, Perth Children’s Hospital, Perth, WA Australia; 2 Neonatal Intensive Care Unit, King Edward Memorial Hospital for Women, Perth, WA Australia; 3 School of Medicine, University of Western Australia, Crawley, WA Australia; 4 Centre for Marine Science and Innovation at the University of New South Wales (UNSW), Sydney, NSW Australia; 5 School of Chemical and Biomedical Engineering, Nanyang Technological University, Singapore, Singapore; 6 Department of Microbiology, PathWest Laboratory Medicine, Perth, WA Australia; 7 Department of Paediatric Surgery, Perth Children’s Hospital, Perth, WA Australia; 8 Department of Genomic and Applied Microbiology, University of Göttingen, Göttingen, Germany

## Abstract

**Abstract:**

Our pilot RCT found that probiotic supplementation with the three-strain bifidobacterial product (*B. breve* M-16V, B. *longum* subsp*. infantis* M-63 and B. *longum* subsp*. longum* BB536) attenuates gut dysbiosis, increases stool short-chain fatty acid (SCFA) levels and improves the growth of head circumference in neonates with congenital gastrointestinal surgical conditions (CGISC). In this article, we have provided guidelines for designing future multicentre RCTs based on the experience gained from our pilot RCT. The recommendations include advice about sample size, potential confounders, outcomes of interest, probiotic strain selection, storage, dose, duration and microbial quality assurance, collection of stool samples, storage and analysis and reporting. Following these guidelines will increase the validity of future RCTs in this area and hence confidence in their results.

**Impact:**

- Probiotic supplementation attenuates gut dysbiosis, increases stool short-chain fatty acid (SCFA) levels and improves the growth of head circumference in neonates with congenital gastrointestinal surgical conditions.
- The current review provides evidence-based guidelines to conduct adequately powered RCTs in this field.

## Background

Our pilot randomised controlled trial (RCT)^1^ found that supplementation with the three-strain probiotic containing *Bifidobacterium breve* M-16V, *Bifidobacterium longum* subsp*. infantis* M-63 and *Bifidobacterium longum* subsp. *longum* BB536 attenuates gut dysbiosis and increases faecal short-chain fatty acid (SCFA) levels in neonates with congenital gastrointestinal surgical conditions (CGISCs). The head circumference growth was better in the probiotic group. While the results were in favour of probiotics, there were areas of uncertainty due to the following reasons:

- Caesarean section^2,3^ rates were higher in the placebo group (70 versus 40%), whereas the incidence of congenital diaphragmatic hernia was higher in the probiotic group (23 versus 3%).
- The relative abundance of proteobacteria, considered the signature of dysbiosis,^4^ was not significantly different between the two groups after 2 weeks of supplementation (22.7% in probiotic versus 50.3% in placebo; *p* = 0.27).
- Being a pilot RCT, the study was not powered to identify clinically significant differences between the groups.

Based on the experience gained from our pilot RCT,^1^ we make the following suggestions for designing future RCTs to overcome the above uncertainties. Some of the suggestions are general and could be used for probiotic RCTs in other population such as preterm infants for prevention of necrotising enterocolitis (NEC).

## Sample size

Our pilot RCT found that the head circumference growth was better in the probiotic group.^1^ Hence, one could hypothesise that the probiotic supplemented group will have better neurodevelopmental outcomes. Our retrospective study^5^ with a sample size of 400 found the incidence of suboptimal neurodevelopmental outcomes to be 16% in term and near-term infants with CGISC. A sample size of 516 infants (258 in each arm) will be required to have 80% power at the two-sided 5% significance level to detect a 50% difference in the primary outcome (16% in controls and 8% in the probiotic group). Since nearly 30% of infants are expected to be discharged home before completing 2 weeks of supplementation, the sample size should be increased by another 154, and hence the final size will be 670 infants. The involvement of multiple centres will be crucial to achieving this sample size within a reasonable time period.

The results of our pilot RCT^1^ also provide the data for estimating the sample sizes for RCTs primarily aimed to compare the stool bacterial community structures between the probiotic and placebo groups. The SIMR package^6^ allows users to calculate sample sizes and conduct power analysis for longitudinal studies. For studies comparing beta-diversity, micropower package^7^ can be used as it calculates sample sizes using pairwise distance and permutational multivariate analysis of variance. It is essential to involve bioinformaticians to ensure appropriate methods for power and sample size calculations and plan the statistical techniques for analyses based on the study hypothesis and design.^8–10^

## Inclusion criteria

Our pilot RCT^1^ focussed on late preterm and term infants with CGISC to avoid the confounding effect of extreme prematurity on gut microbiota. Future RCTs, if of adequate sample size, could include even very preterm (<32 weeks) and extremely preterm (<28 weeks) infants with CGISC. With a large sample size, baseline characters, including gestational age, are expected to be similar between the study groups. Probiotic supplementation is safe and beneficial even in extremely preterm infants without surgical conditions and reduces the risk of NEC, feed intolerance, and late-onset sepsis.^11–15^

## Randomisation

Since our pilot RCT^1^ had a small sample size of 60 infants, we used computer-generated random sequence numbers in random blocks of two and four to ensure that an equal number of infants receive probiotics or a placebo. However, this method did not minimise the chance of unequal distribution of essential confounders (e.g. mode of delivery and severity of the surgical condition), which can affect the gut microbiota.

Therefore, future studies should consider using “treatment allocation by minimisation”^16^ or “rank minimisation”^17^ to achieve balance, especially if the sample size is small. The involvement of clinical epidemiologists at a very early stage of trial protocol is crucial to achieving the ideal design for the trial, including the randomisation method.

Factors such as gestational diabetes,^18^ mode of delivery,^3^ intrapartum antibiotics^19–21^ and maternal probiotics,^22–24^ gestational age^25^ and underlying surgical conditions that can affect the neonatal gut microbiota need to be balanced between the probiotic and placebo groups. Since the type of milk used (breast milk versus cow’s milk-based versus hydrolysed formula) can influence gut microbiota,^26^ standardising their feeding regimen is desirable but unlikely to be feasible, given that multiple factors can affect milk production and mother’s choice and clinicians’ opinions.

## Selection of probiotic product

So far, only two pilot RCTs have evaluated probiotics in neonates with CGSC. The first study was by Powell et al., who reported that daily administration of the single-strain probiotic *B.* *longum* subsp*. infantis* (2 × 10^9^ colony-forming unit (CFU) per day) resulted in partial attenuation of gut dysbiosis in neonates with gastroschisis.^27^ The second RCT was ours,^1^ in which the multi-strain probiotic product, a mixture of *B.* *breve* M-16V, *B.* *longum* subsp*. infantis* M-63 and *B*. *longum* subsp. *longum* BB536 (1 × 10^9^ of each strain per sachet; Morinaga Milk Industry Co, Japan) was safe and effective in improving gut microbiota and SCFA levels. We found the study sachets (probiotics and placebo) free of harmful bacteria by conducting regular microbial analyses on random sachets. Future RCTs in neonates with CGISC could consider using this product or the strain used by Powell et al.^27^ In the other small RCT currently being conducted in Canada (ClinicalTrials.gov identifier NCT03266315), 20 newborn infants with CGSCs will be randomised to receive the multi-strain product FloraBaby^TM^, a mixture of bifidobacteria and lactobacilli strains. It is essential to wait for the results of that study before conducting multicentre RCTs using that product in neonates with CGISC.

While many other probiotic products are safe and effective in preterm infants without surgical conditions, it is essential to test them in pilot RCTs in neonates with CGISCs before considering them in large multicentre RCTs. This is because, unlike preterm infants without CGISCs, neonates with CGISC undergo contrast dye studies, laparotomies, get exposed to general anaesthetics and some of them receive bowel enemas/suppositories, all of which could influence the effects of the administered probiotics.

Another factor to consider is the safety of supplemented probiotics, especially the potential for translocation and causing sepsis. Unlike preterm infants without surgical problems, neonates with CGISC undergo laparotomy. Hence, there is a risk that the administered live probiotic bacteria could spill over into the peritoneal cavity and systemic circulation resulting in sepsis and seeding in various organs. While infants in our pilot RCT^1^ or Powell et al.^27^ did not experience such a complication, a large sample size study would be needed to ensure safety.

In that context, it is also reassuring that infection due to administered probiotic organisms is infrequent even among extremely preterm infants and can be treated with antibiotics. Sakurai et al.^28^ reported that bifidobacterial bacteraemia occurred in 6 out of 298 neonates (i.e. 2%), but none had severe illnesses due to bacteraemia. They speculated that the reason behind the high incidence of *B. breve* bacteraemia in their cohort was rigorous laboratory methods.

Alternatives to probiotics such as prebiotics^29,30^ and para-probiotics (dead probiotic bacteria)^31^ that are unlikely to carry the risk of probiotic sepsis should also be tested in future RCTs.

## Dose of probiotic supplements

To our knowledge, there are no dose-finding studies in neonates with surgical conditions. There are two studies evaluating the dose response^32,33^ and one study on dose interval^34^ of probiotics on colonisation rates in preterm infants.

Dutta et al.^32^ randomly allocated 149 preterm infants to groups A–D (received 12-hourly probiotic supplements of 10^10^ cells for 21 days, 10^10^ cells for 14 days, 10^9^ cells for 21 days and placebo, respectively). They reported that colonisation with Lactobacillus and Bifidobacterium by day 28 was significantly higher in groups A, B, and C versus placebo, respectively. They also reported that there were trends toward more CFU of Lactobacillus and Bifidobacterium per ml of stool in group A versus B and group B versus C.

Underwood et al.^33^ randomly allocated 12 preterm infants receiving formula feedings to receive either *B. infantis* or *B. lactis* in increasing doses over a 5-week period. The dose was 5 × 10^7^, 1.5 × 10^8^, 4.5 × 10^8^, 1.4 × 10^9^, 4.2 × 10^9^, at weeks 1, 2, 3, 4 and 5, respectively. There was a greater increase in faecal bifidobacteria among infants receiving *B. infantis* than those receiving *B. lactis*. This difference was most marked at a dose of 1.4 × 10^9^ CFU twice daily. Relative abundance of bifidobacteria declined with increasing dosage over time/dose in the *B. lactis* group and showed a statistically nonsignificant trend towards increase in the *B. infantis* group. It shows that the colonisation response is not only dependent on the dose but also on the strain.

Watkins et al.^34^ investigated the appropriate dosing interval of a dual strain probiotic given daily (*n* = 8), biweekly (*n* = 8) and weekly (*n* = 10) in preterm infants <32 weeks’ gestation. The control group consisted of 12 preterm infants who did not receive the probiotic. *Bifidobacterium bifidum* (1 × 10^9^ CFU) and *Lactobacillus acidophilus* (1 × 10^9^ CFU), was administered (2 × 10^9^ CFU of bacteria in total), until 34 weeks postmenstrual age (PMA). Stool samples were collected at 31, 34, 41 and 44 weeks PMA. At all ages assessed, colonisation levels of administered probiotic organisms were higher in the once daily group. They concluded that a daily dose is a suitable dosage for preterm infants.

Given that there is insufficient evidence regarding the dose of probiotics, dose-finding studies with the chosen strain/s need to be conducted prior to embarking on large RCTs.

Our study^1^ used a total of 3 billion probiotic organisms per day (3 × 10^9^ CFU), whereas Powell et al.^27^ had used 2 billion probiotic organisms per day (2 × 10^9^ CFU). Powell et al.^27^ reported partial attenuation of gut dysbiosis after probiotic supplementation, whereas our study showed a higher attenuation level. Hence, it provides indirect evidence that a dose of at least 3 × 10^9^ CFU per day could be used in future RCTs. Doses higher than 3 × 10^9^ CFU per/day may offer further benefits but need to be tested in dose-finding studies initially (for example, 3 × 10^9^ versus 4 × 10^9^ versus 5 × 10^9^ versus 6 × 10^9^). Smaller quantities <1 billion (i.e. <1 × 10^9^) CFU may not be enough to colonise the gut adequately, limiting their effectiveness.^35,36^

## Storage of probiotics/placebo sachets

We stored the main stock of probiotic/placebo sachets in the trial pharmacy department in the refrigerator at 2–8 °C. To enable ease of access, at least five such sequentially numbered boxes were kept in the automated dispensing machine (ADM) within the neonatal intensive care unit (NICU) at refrigerator temperatures of 2–8 °C. Once parental consent was obtained, the box next in order was labelled with the Unique Medical Record Number sticker of that infant, thereby declaring that particular package belongs to that infant for the entire hospital stay. Each day, one sachet from that box was taken out from the ADM by the nurses and administered to that particular infant. A similar approach could be undertaken in future RCTs. Keeping the trial supplements at room temperatures may affect their viability and longevity.

## Timing of starting the trial supplements

In our study,^1^ trial supplements were commenced predominantly in the immediate postoperative period. The main reason for the delay was difficulty collecting baseline stool samples for reasons discussed earlier. In our RCT and the RCT by Powell et al., probiotics were commenced in the immediate post-operative period and found it to attenuate dysbiosis. The majority of the studies involving adults who underwent gut surgeries found benefits of probiotics when administered in the preoperative period.^37^ To obtain maximum benefit, the best time to commence probiotics is probably in the pre-operative period, but collection of stool samples before starting probiotics may not be feasible in all cases.

## Administration of probiotics while infants are fed nil-enterally

We administered the study supplements even when the infants were fed nil-enterally and found no side effects.^1^ Waiting until enteral feeds are commenced may decrease the efficacy of probiotics by delaying their gut colonisation. Considering the small dose volume and low osmolarity (320–350 mOsm/l) when reconstituted in expressed breast milk,^38^ it is reasonable to start the supplement even if the infant is fed nil-enterally.

## When to withhold supplementation

In our study,^1^ we continued supplementation even when infants were critically ill as long as there were no significant abdominal symptoms. We suggest that supplements only be withheld if there is a gut perforation or suspicion of abdominal compartment syndrome with a distended, firm and tender abdomen to minimise the risk of gut translocation by the supplemented probiotic organisms.

## Care immediately after administering the trial supplements

We administered the trial medication as a single daily dose for convenience. Many infants with CGISC will have nasogastric tubes (NGTs) on free drainage or suction for gastric decompression. We clamped the NGT for at least 1 h and preferably 3–4 h to prevent the retrograde flow of the administered probiotics/placebo into the free-drain container.^27^

## Hand hygiene precautions

Rigorous hand hygiene needs to be followed to prevent the risk of cross-contamination. While our pilot RCT^1^ did not specifically address the issue of cross-contamination (aka cross-colonisation),^39^ we were reassured that the relative abundance of the genus *Bifidobacterium* in the placebo group was only about 5% at all time points T2–T4. In contrast, it was around 35–45% in the probiotic group. Hence, even if there was cross-contamination, the load was not enough to allow them to colonise adequately and, therefore, unlikely to be clinically significant.

The relative abundance of 5% for the genus *Bifidobacterium* in the placebo arm after 2 weeks of supplementation and also prior to discharge in our study^1^ was lower than the Australian PROPREMS trial in preterm infants, in which it was 17.5% (SD 27.4) in the placebo group and 36.4% (32.5) in the probiotic group.^40^ The UK PIPS trial^41^ in preterm infants (non-surgical) reported high cross-colonisation rates because 49% of infants in the placebo group were colonised (culture positive) with the administered strain. However, they did not report on the relative abundances, and hence it is difficult to know if such cross-colonisation impacted the clinical outcomes of the trial.^41^ Future studies should report cross-colonisation rates and relative abundances from study infants.

NICU environmental contamination (refrigerator doors, telephone receivers, infant cots, monitors) with the administered probiotic organism is possible and may result in cross-colonisation of infants receiving placebo.^39,42^ Further research is needed to confirm if such risk may be lessened if the preparation is done off-site from the NICU environment.

Some researchers have recommended that future multicentre studies may have to adapt a cluster RCT design to overcome the issue of cross-contamination.^41^ Irrespective of whether the trial design is conventional or a cluster RCT and whether the supplements are prepared in the NICU are off-site, strict hand hygiene is essential while handling them and caring for neonates.

## Medication reconciliation

Our method was to record doses administered, omitted and wasted sachets. It was matched against the number of unused sachets in the package after it was collected by the trial pharmacists when the infant had completed the intervention.

## Safety of probiotics

Researchers need to inform the parents and research ethics committees that probiotics are live bacterial organisms, and there are reports of sepsis due to the administered probiotic organism.^43–49^ However, researchers should also reassure parents that most cases of probiotic sepsis were successfully treated by antibiotics.^28,43^ In addition, it is essential to inform parents that 63 RCTs, 30 observational studies and many meta-analyses including the Cochrane review have found probiotics to be safe in preterm (non-surgical) infants.^11–15,35,50–56^ Our pilot RCT^1^ and the RCT by Powell et al.^27^ found probiotics to be safe in neonates with CGISC. The only published case report of mortality after probiotic supplementation in a preterm (non-surgical) infant was because of contamination of the product.^57^ Independent assessment of the *product quality* is of paramount importance,^58^ and probiotics should be free of contaminants and from companies with a high safety track record.

## Ongoing microbiological quality assurance

Having a well-resourced microbiology laboratory is essential for any centre planning to conduct RCT of probiotics.^58^ It is crucial to conduct microbial analysis of random sachets of study supplements to rule out the presence of harmful pathogens and to check viability and colony counts of the probiotic strain. All routine clinical specimens (blood, urine, cerebrospinal fluid, endotracheal secretions, wound swabs) from study infants should be analysed using culture methods to enable diagnosis of infections due to supplemented probiotic organisms. As per our standard practice for routine clinical care, we used the Becton Dickinson BACTEC™ PEDS PLUS™/F Medium aerobic blood culture bottles with incubation monitored in the Bactec 9120 system.^59^ No special culture bottles were used for the study. Our laboratory’s automated blood culture system detects bifidobacteria (if present) within the standard 5-day (120 h) incubation period. While some studies have shown that, if incubation in aerobic culture bottles is ceased after 120 h, a few cases of bifidobacterial bacteraemia could be missed,^28^ we decided to restrict to 120 h because going beyond that period would require larger capacity size incubators. The other issue if incubation goes beyond 120 h is the likely recovery of slow-growing contaminant organisms, which can affect the clinical interpretation of the results. On the other hand, incubation of <120 h will miss many cases and hence cannot be recommended.

## Collection of stool samples

In our study,^1^ we collected stool samples into 0.5 ml sterile micro-tubes (sarstedt.com). If the sample is collected in a different container and subsequently transferred to the micro-tubes, there is a risk of microbial contamination. We used sterile wooden spoons to scoop fresh samples from the nappies (diapers) of study infants. There were many challenges during the collection of stool samples. (a) Since the nappies are checked only once in 3–4 h, in many cases, stools had dried up by then. (b) Watery stools got absorbed into the nappies, so the collection was impossible. (c) Delayed passage of meconium and infrequent stooling in the pre-operative and immediate post-operative periods due to intestinal obstruction, postoperative ileus, and the use of morphine/fentanyl. (d) Use of radio-contrast enema or upper GI contrast for diagnostic purposes. If stool samples are collected after the contrast study, it will not represent the actual gut microbiota of the infant. (e) Missed opportunity to collect samples because most infants pass only one stool in the pre-operative period and none until 3–5 days in the post-operative period. Hence, extra vigilance and cooperation by the bedside nurses are essential to ensure the timely collection of stool samples.

A recent study found rectal swabs correlated well with the simultaneously collected faecal samples in neonates.^60^ In contrast, a similar study in critically ill adults reported systematic differences in gut microbial profiles between simultaneously collected rectal swabs and stool samples.^61^ Further studies are needed to confirm the reliability of rectal swabs for microbial analysis. Such studies should also evaluate the effect of collection mode on stool SCFA levels.

## Labelling of stool samples

Accurate labelling of the micro-tubes is essential to maintain the integrity of the data. They could be labelled the samples as follows if the total sample size is 100–999:

The first three digits to represent the study number of the infant, the second two digits refer to the sample number and subsequent alphabets represent the purpose of the sample.

Example: 003-01-DNA means study infant number 3, first stool sample (i.e. before commencing supplements), and the sample is for DNA sequencing.

003-01-SCFA means study infant number 3, stool sample before commencing supplements, and the sample is for SCFA analysis.

## Storage of stool samples

In our study,^1^ we stored the samples in a −20 °C freezer immediately after collection and subsequently transferred them in a cold portable cooler for final storage at −80 °C within next 96 h. While rapid freezing to −80 °C is considered the best practice, it is not feasible even in the most resourceful settings. On the other hand, storing the samples at room temperatures is not recommended as it is known to lower Shannon diversity and evenness.^62^ It is suggested that the samples should be preserved at −20 °C within 15 min after collection and then transferred on dry ice within 24 h of collection and stored at −80 °C thereafter.^10^

Recent studies have shown that specific commercially available reagents allow for stool samples collection, preservation and storage at ambient temperatures for longer periods.^63^ Many laboratories provide their vials to collect stool samples that have DNA-preservation agents in the vials. Hence, it is important to discuss with the laboratory at the protocol stage of the RCT. It is also essential to ensure that the sample preservation and storage methods are consistent across all samples to minimise variations in results.^10,62,64^

## Shipping of stool samples

Given that stool samples are biological specimens, only accredited couriers should be used for shipping such samples. Maintaining a cold chain at −80 °C using dry ice is essential, especially while sending the samples that do not contain preservation media. Transportation logistics, including temperatures, need to be discussed with the receiving laboratory well in advance.

## Method of analysing stool samples for gut microbial data

There are excellent guidelines on the best approaches for analysing microbial data.^65^ Briefly, the methods used in microbiome research include amplicon, metagenomic and metatranscriptomic sequencing. The amplicon sequencing involves the 16S rRNA gene sequencing for bacteria. It is relatively inexpensive, but the analysis is limited to genus-level taxonomic resolution. On the other hand, the metagenomic sequencing method sequences all microbial genomes (DNA) within a sample. It extends the taxonomic resolution to species or strain level. If there is adequate funding, metagenomic sequencing is preferred. Metatranscriptomics uses RNA sequencing to profile transcription in microbiomes, providing information on gene expression and the active functional output of the microbiome. It gives better insight into the functional activity of a microbial community. The pros and cons of each method are well described by Knight et al.^65^ It is important to decide whether to limit to 16s ribosomal RNA gene sequencing or metagenomics during the early stages of protocol development in collaboration with the laboratory scientists. Even if the aim is to restrict to the former, it is helpful to collect additional stool samples and store them at −80 °C so that metagenomic sequencing can be undertaken in future when funding becomes available.

## Method of analysing stool samples for SCFA

SCFAs are produced mainly by intestinal microbiota and play an important role in many biological processes in humans. Gas chromatography–mass spectrometry^66–68^ is the commonly used method for SCFA assay. Alternative methods are high-performance liquid chromatography, nuclear magnetic resonance and capillary electrophoresis.^69^

Since SCFAs are volatile, keeping the stool samples in appropriate conditions after collection is important. Samples are usually kept at −80 °C, although many researchers have successfully used −20 °C.^69^ It is important to screw the lid tight and not open it until it reaches the laboratory for analysis. The stool samples for SCFA analysis should be collected in vials separate from those used for microbial analysis.

## Ensuring blinding of the data

It is important to ensure blinding of the group allocation till the full results are available. Only the trial pharmacist or a similar professional with no vested interest in the project should know which sachets are probiotic and placebo. When the stool samples are sent to laboratories for microbial and SCFA analyses, they should be labelled as groups 1 and 2 to enable comparison without disclosing the groups. Clinical data also should be collected and compared as group 1 and group 2. Only in the end, unblinding should be done by the trial pharmacist.

Once the analysis comparing the microbiota of group 1 versus group 2 is completed, the bioinformatician may be able to guess group allocations (even when blinded) if the relative abundance of the supplemented bacteria is higher in one group. If researchers and statisticians assessing clinical outcomes become aware of those results, bias might be introduced. Hence statistical analysis of clinical data must be done by people who are blinded to the results of the microbial analysis and vice versa. This is especially important when the primary outcome of interest is clinical (sepsis, mortality, duration of hospital stay, time to full feeds, neurodevelopment).

## Data safety and monitoring board (DSMB)

Establishing a DSMB is essential before recruitment into the RCT.^70^ The charter should have pre-defined stopping rules both for efficacy and safety. While *p* values around stopping rules are essential, they should not be the sole criteria while deciding whether the trial should continue or stop.

## Reporting

Reporting metagenomic analysis of stool samples should follow the recently published STROBE-Metagenomics guidelines.^71^ Given that the study design will be an RCT, CONSORT guidelines help report clinical outcomes.

## Conclusions

In summary, following these guidelines will increase the validity of future RCTs in this area, hence confidence in their results.

## Data Availability

Data sharing is not applicable to this article as no data sets were generated or analysed during the current study.
